# Supplementary material for: Prevalence of SARS-CoV-2 antibodies in France: results from nationwide serological surveillance
Source: Nat Commun. 2021 May 21;12:3025. doi: 10.1038/s41467-021-23233-6 (PMC8140151; doi:10.1038/s41467-021-23233-6)
Supplement: Supplementary file 1 — Supplementary Information [file 41467_2021_23233_MOESM1_ESM.pdf]

# Supplementary Information

*Supplementary Table 1: Calibration of LuLISA N, LuLISA S and pseudo-neutralisation assays on prepandemic samples and confirmed COVID-19 cases.*

| | Serological Assay | | | |
|---|---|---|---|---|
| | **LuLISA N** | **LuLISA S** | **PNT** | **LN\|LS\|PNT** |
| Readings threshold (RLU/s) | 39915 | 27821 | 14037 | as defined at left |
| Prepandemic samples | 523 | 232 | 223 | 223 |
| Confirmed COVID-19 cases | 309 | 45 | 99 | 45 |
| False positive | 0 | 0 | 0 | 0 |
| True positive | 266 | 43 | 84 | 45 |
| True negative | 523 | 232 | 223 | 223 |
| False negative | 43 | 2 | 15 | 0 |
| Specificity | 1·00 | 1·00 | 1·00 | 1·00 |
| Sensitivity | 0·86 | 0·96 | 0·85 | 1·00 |

LN=LuLISA N assay, LS=LuLISA S assay, PNT=Pseudo-neutralisation assay. LN|LS|PNT refers to positive in any of the three assays. Readings are expressed in relative light units per second (RLU/s). Confirmed COVID-19 cases were hospitalised patients with positive RT-PCR for SARS-CoV-2.

*Supplementary Table 2: Distribution of sample specimens included by collection period.*

| | 9-15 March 2020 | | 6 -12 April 2020 | | 11-17 May 2020 | | France | |
|---|---|---|---|---|---|---|---|---|
| | n | % | n | % | n | % | n | % |
| Overall | 3834 | 100 | 3595 | 100 | 3592 | 100 | 66784232 | 100 |
| Sex | | | | | | | | |
| Male | 1689 | 44.1 | 1557 | 43.3 | 1543 | 43 | 32263970 | 48.3 |
| Female | 2145 | 55.9 | 2038 | 56.7 | 2049 | 57 | 34520262 | 51.7 |
| Age | | | | | | | | |
| 0-9 | 359 | 9.4 | 167 | 4.6 | 255 | 7.1 | 7670792 | 11.5 |
| 10-19 | 420 | 11 | 298 | 8.3 | 336 | 9.4 | 8263694 | 12.4 |
| 20-29 | 423 | 11 | 355 | 9.9 | 318 | 8.9 | 7434257 | 11.1 |
| 30-39 | 500 | 13 | 461 | 12.8 | 409 | 11.4 | 8250560 | 12.4 |
| 40-49 | 487 | 12.7 | 455 | 12.7 | 390 | 10.9 | 8556317 | 12.8 |
| 50-59 | 461 | 12 | 489 | 13.6 | 469 | 13.1 | 8770338 | 13.1 |
| 60-69 | 455 | 11.9 | 487 | 13.5 | 496 | 13.8 | 7991860 | 12 |
| 70-79 | 382 | 10 | 455 | 12.7 | 480 | 13.4 | 5690626 | 8.5 |
| 80+ | 347 | 9.1 | 428 | 11.9 | 439 | 12.2 | 4155788 | 6.2 |
| Region | | | | | | | | |
| Guadeloupe | 262 | 6.8 | 149 | 4.1 | 252 | 7 | 376879 | 0.6 |
| Martinique | 111 | 2.9 | 208 | 5.8 | 254 | 7.1 | 358749 | 0.5 |
| Guyane | 96 | 2.5 | 64 | 1.8 | 102 | 2.8 | 290691 | 0.4 |
| La Réunion | 144 | 3.8 | 90 | 2.5 | 105 | 2.9 | 859959 | 1.3 |
| Île-de-France | 641 | 16.7 | 614 | 17.1 | 534 | 14.9 | 12278210 | 18.4 |
| Centre-Val-de-Loire | 146 | 3.8 | 116 | 3.2 | 122 | 3.4 | 2559073 | 3.8 |
| Bourgogne-Franche Comté | 102 | 2.7 | 138 | 3.8 | 123 | 3.4 | 2783039 | 4.2 |
| Normandie | 208 | 5.4 | 210 | 5.8 | 208 | 5.8 | 3303500 | 4.9 |
| Hauts-de-France | 301 | 7.9 | 309 | 8.6 | 262 | 7.3 | 5962662 | 8.9 |
| Grand-Est | 255 | 6.7 | 199 | 5.5 | 205 | 5.7 | 5511747 | 8.3 |
| Pays de la Loire | 119 | 3.1 | 156 | 4.3 | 135 | 3.8 | 3801797 | 5.7 |
| Bretagne | 129 | 3.4 | 121 | 3.4 | 95 | 2.6 | 3340379 | 5 |
| Nouvelle-Aquitaine | 339 | 8.8 | 328 | 9.1 | 282 | 7.9 | 5999982 | 9 |
| Occitanie | 281 | 7.3 | 250 | 7 | 206 | 5.7 | 5924858 | 8.9 |
| Auvergne-Rhône-Alpes | 233 | 6.1 | 286 | 8 | 234 | 6.5 | 8032377 | 12 |
| Provence-Alpes-Côte d'Azur | 453 | 11.8 | 296 | 8.2 | 397 | 11.1 | 5055651 | 7.6 |
| Corse | 14 | 0.4 | 61 | 1.7 | 76 | 2.1 | 344679 | 0.5 |

All available residual sera were included from French overseas regions (Guadeloupe, Martinique, French Guiana and La Reunion). Sample selection was stratified by age, sex and region for Metropolitan France.

*Supplementary Table 3: Combination of unadjusted results for the three serological assays by collection period.*

| **LuLISA N** | **LuLISA S** | **PNT** | **9-15 March 2020** | | **6-12 April 2020** | | **11-17 May 2020** | |
|---|---|---|---|---|---|---|---|---|
| | | | **n** | **%** | **n** | **%** | **n** | **%** |
| positive | positive | positive | 1 | 0.0 | 66 | 1.8 | 87 | 2.4 |
| positive | positive | negative | 0 | 0.0 | 13 | 0.4 | 6 | 0.2 |
| positive | negative | positive | 0 | 0.0 | 5 | 0.1 | 3 | 0.1 |
| negative | positive | positive | 0 | 0.0 | 9 | 0.3 | 13 | 0.4 |
| positive | negative | negative | 6 | 0.2 | 18 | 0.5 | 35 | 1.0 |
| negative | positive | negative | 20 | 0.5 | 37 | 1.0 | 32 | 0.9 |
| negative | negative | positive | 0 | 0.0 | 8 | 0.2 | 2 | 0.1 |
| negative | negative | negative | 3807 | 99.3 | 3439 | 95.7 | 3414 | 95.0 |
| | | | 3834 | 100 | 3595 | 100 | 3592 | 100 |

Positivity thresholds in relative light units per second (RLU/s) are 39915 for LuLISA N, 27821 for LuLISA S and 14037 for Pseudo-neutralisation (PNT).

*Supplementary Table 4: Estimated prevalence of SARS-CoV-2 antibodies in French population from March-May 2020.*

| | **9-15 March 2020** | **6 -12 April 2020** | **11-17 May 2020** |
|---|---|---|---|
| | Prevalence (95% CI) | Prevalence (95% CI) | Prevalence (95% CI) |
| Overall | 0·4% (0·1-0·9) | 4·1% (3·3-5·0) | 4·9% (4·0-5·9) |
| Sex | | | |
| Male | 0·5% (0·1-1·0) | 4·5% (3·5-5·6) | 5·4% (4·3-6·6) |
| Female | 0·4% (0·0-0·8) | 3·8% (2·9-4·7) | 4·5% (3·6-5·5) |
| Age group, years | | | |
| 0-9 | 0·2% (0·0-0·6) | 2·3% (0·9-4·1) | 2·7% (1·1-4·9) |
| 10-19 | 0·4% (0·0-0·8) | 3·7% (2·3-5·3) | 4·4% (2·9-6·2) |
| 20-29 | 0·4% (0·1-1·0) | 4·3% (3·0-6·0) | 5·2% (3·6-7·1) |
| 30-39 | 0·4% (0·0-0·8) | 3·7% (2·4-5·1) | 4·4% (2·9-6·0) |
| 40-49 | 0·5% (0·1-1·1) | 4·9% (3·4-6·7) | 5·9% (4·2-7·9) |
| 50-59 | 0·5% (0·1-1·1) | 5·1% (3·7-6·8) | 6·1% (4·4-8·0) |
| 60-69 | 0·5% (0·1-1·1) | 5·1% (3·6-6·8) | 6·0% (4·4-8·1) |
| 70-79 | 0·4% (0·0-0·9) | 3·8% (2·6-5·2) | 4·5% (3·1-6·1) |
| ≥80 | 0·4% (0·0-0·9) | 4·1% (2·9-5·5) | 4·8% (3·4-6·5) |
| Regions | | | |
| Guadeloupe | 0·3% (0·0-0·7) | 2·7% (1·4-4·4) | 3·3% (1·7-5·1) |
| Martinique | 0·2% (0·0-0·5) | 2·0% (1·0-3·4) | 2·4% (1·2-3·9) |
| French Guiana | 0·6% (0·1-1·4) | 6·0% (3·3-9·9) | 7·1% (4·0-11·5) |
| La Reunion | 0·3% (0·0-0·8) | 2·5% (0·8-4·9) | 3·0% (1·0-5·8) |
| Île-de-france | 0·8% (0·1-1·6) | 7·4% (5·7-9·4) | 8·8% (6·9-11·0) |
| Centre-Val-de-Loire | 0·3% (0·0-0·7) | 2·6% (1·1-4·6) | 3·1% (1·3-5·4) |
| Bourgogne-Franche Comté | 0·4% (0·0-0·9) | 3·6% (1·9-5·8) | 4·3% (2·2-6·9) |
| Normandie | 0·3% (0·0-0·8) | 3·3% (1·9-5·1) | 3·9% (2·3-6·0) |
| Hauts-de-France | 0·3% (0·0-0·7) | 2·9% (1·7-4·4) | 3·5% (2·1-5·3) |
| Grand-Est | 0·7% (0·1-1·7) | 7·2% (4·8-10·2) | 8·6% (5·8-11·8) |
| Pays de la Loire | 0·3% (0·0-0·7) | 2·9% (1·4-4·9) | 3·5% (1·8-5·9) |
| Bretagne | 0·2% (0·0-0·6) | 2·1% (0·8-4·0) | 2·6% (1·0-4·8) |
| Nouvelle-Aquitaine | 0·3% (0·0-0·6) | 2·6% (1·5-4·0) | 3·2% (1·8-4·7) |
| Occitanie | 0·3% (0·0-0·6) | 2·6% (1·3-4·1) | 3·1% (1·6-4·9) |
| Auvergne-Rhône-Alpes | 0·4% (0·0-0·8) | 3·7% (2·3-5·4) | 4·4% (2·8-6·5) |
| Provence-Alpes-Côte d'Azur | 0·3% (0·0-0·6) | 2·8% (1·6-4·2) | 3·3% (2·0-4·9) |
| Corse | 0·3% (0·0-0·8) | 3·2% (1·3-5·8) | 3·8% (1·5-6·9) |

Prevalence of SARS-CoV-2 antibodies was based on at least one positive test among LuLISA S, LuLISA N and pseudo-neutralisation assays (see main text).

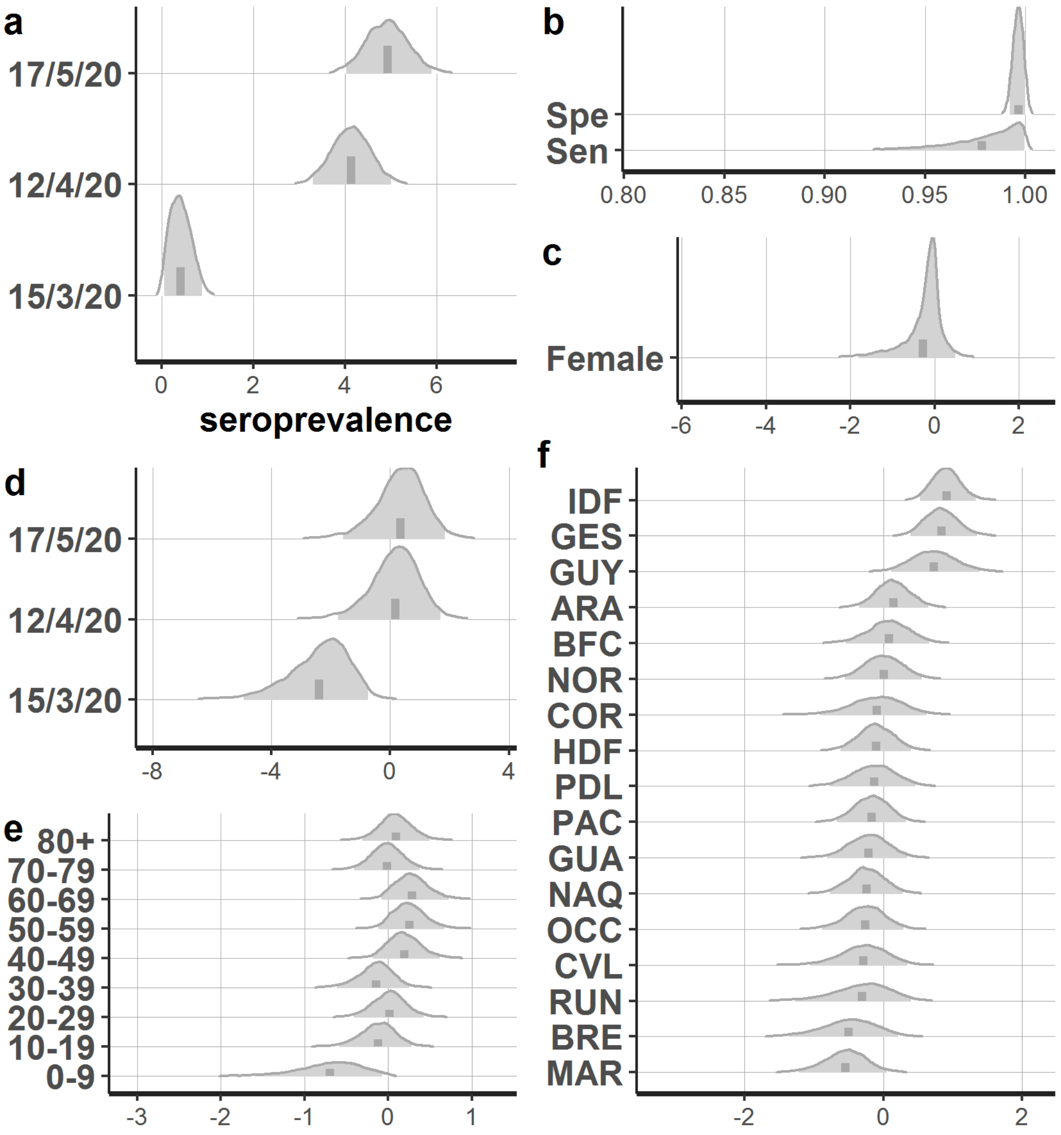


*Supplementary Figure 1: Posterior distributions of prevalence, test performance and regression coefficients.*

**a** Seroprevalence by study period . **b** Overall sensitivity and specificity. Regression coefficients for study period (**c**), sex (**d**), age class (**e**) and region (**f**). 90% uncertainty intervals (combining all chains) are in light grey and point estimates of mean in darker grey.
