# Supplementary material for: Prevalence of SARS-CoV-2 antibodies in France: results from nationwide serological surveillance
Source: Nat Commun. 2021 May 21;12:3025. doi: 10.1038/s41467-021-23233-6 (PMC8140151; doi:10.1038/s41467-021-23233-6)
Supplement: Supplementary file 3 — Reporting Summary [file 41467_2021_23233_MOESM3_ESM.pdf]

nature research

Corresponding author(s): Stéphane Le Vu

Last updated by author(s): Mar 8, 2021

Reporting Summary

Nature Research wishes to improve the reproducibility of the work that we publish. This form provides structure for consistency and transparency in reporting. For further information on Nature Research policies, see our Editorial Policies and the Editorial Policy Checklist.

Statistics

For all statistical analyses, confirm that the following items are present in the figure legend, table legend, main text, or Methods section.

n/a	Confirmed	
[ ]	[x]	The exact sample size (*n*) for each experimental group/condition, given as a discrete number and unit of measurement
[ ]	[x]	A statement on whether measurements were taken from distinct samples or whether the same sample was measured repeatedly
[x]	[ ]	The statistical test(s) used AND whether they are one- or two-sided<br>*Only common tests should be described solely by name; describe more complex techniques in the Methods section.*
[ ]	[x]	A description of all covariates tested
[ ]	[x]	A description of any assumptions or corrections, such as tests of normality and adjustment for multiple comparisons
[ ]	[x]	A full description of the statistical parameters including central tendency (e.g. means) or other basic estimates (e.g. regression coefficient) AND variation (e.g. standard deviation) or associated estimates of uncertainty (e.g. confidence intervals)
[x]	[ ]	For null hypothesis testing, the test statistic (e.g. *F*, *t*, *r*) with confidence intervals, effect sizes, degrees of freedom and *P* value noted<br>*Give P values as exact values whenever suitable.*
[ ]	[x]	For Bayesian analysis, information on the choice of priors and Markov chain Monte Carlo settings
[ ]	[x]	For hierarchical and complex designs, identification of the appropriate level for tests and full reporting of outcomes
[ ]	[x]	Estimates of effect sizes (e.g. Cohen's *d*, Pearson's *r*), indicating how they were calculated

Our web collection on statistics for biologists contains articles on many of the points above.

Software and code

Policy information about availability of computer code

Data collection	Data collection used R 3.6.2.
Data analysis	Data analysis was carried out using R 3.6.2 and the R interface to Stan (RStan 2.21.2). Models were written using Stan language. Reproducible code is publicly available at https://github.com/slevu/serpico2 doi:10.5281/zenodo.4586147

For manuscripts utilizing custom algorithms or software that are central to the research but not yet described in published literature, software must be made available to editors and reviewers. We strongly encourage code deposition in a community repository (e.g. GitHub). See the Nature Research guidelines for submitting code & software for further information.

Data

Policy information about availability of data

All manuscripts must include a data availability statement. This statement should provide the following information, where applicable:

- Accession codes, unique identifiers, or web links for publicly available datasets
- A list of figures that have associated raw data
- A description of any restrictions on data availability

Individual data from the analysis can be made available upon reasonable request to the corresponding author and might require partial aggregation or downsampling to protect patient privacy. A synthetic population dataset is available along with code. The source data underlying Fig. 1 and 2 are provided with this paper as a Source Datafile.

Field-specific reporting

Please select the one below that is the best fit for your research. If you are not sure, read the appropriate sections before making your selection.

- [x] Life sciences
- [ ] Behavioural & social sciences
- [ ] Ecological, evolutionary & environmental sciences

For a reference copy of the document with all sections, see nature.com/documents/nr-reporting-summary-flat.pdf

Life sciences study design

All studies must disclose on these points even when the disclosure is negative.

Sample size	Relying on early modelling of the COVID-19 epidemic in France, which estimated an expected overall prevalence of 3% as of 28 March 2020, we calculated a target sample size of 3,500 per collection period, with a margin of error of 0.55%.
Data exclusions	We excluded samples from Mayotte Island (smallest French overseas region) from the analysis due to an insufficient number of available sera.
Replication	Our findings cannot be replicated using the same study design as serum samples from routine clinical laboratory activity are discarded weekly or monthly. Our study results were obtained from residual sera collected and stored in biobanks and specifically sampled for the purpose of this research.
Randomization	There was no allocation in experimental groups per se in this study. However, we randomly sampled available sera at the biobanks. Sampling was stratified by sex, 10-year age groups (0-9 years to ≥80 years) and region of Metropolitan France to ensure the precision described above.
Blinding	Blinding was not relevant to the study as all specimens were collected for routine clinical purpose, independently of this research and without (intervention) group allocation. No clinical data were collected which might allow a differential treatment by investigators.

Reporting for specific materials, systems and methods

We require information from authors about some types of materials, experimental systems and methods used in many studies. Here, indicate whether each material, system or method listed is relevant to your study. If you are not sure if a list item applies to your research, read the appropriate section before selecting a response.

Materials & experimental systems

n/a	Involved in the study	
☐	☒	Antibodies
☐	☒	Eukaryotic cell lines
☒	☐	Palaeontology and archaeology
☒	☐	Animals and other organisms
☐	☒	Human research participants
☒	☐	Clinical data
☒	☐	Dual use research of concern

Methods

n/a	Involved in the study	
☒	☐	ChIP-seq
☒	☐	Flow cytometry
☒	☐	MRI-based neuroimaging

Antibodies

Antibodies used	Anti-human IgG VhH were developed in-house at the Institut Pasteur. They have been previously described in Anna et al European Journal of Immunology . 2020 Dec 1 doi: 10.1002/eji.202049058; Sterlin et al Science Translational Medecine DOI:10.1126/scitranslmed.abd2223
Validation	Individual test characteristics were assessed using sets of pre-pandemic sera collected before 04/09/2019 and sera from hospitalised cases of COVID-19 confirmed by RT-PCR. Details are given in the methods section and Figure S1.

Eukaryotic cell lines

Policy information about cell lines

Cell line source(s)	ATCC
Authentication	the HEK 293T cell line was not authenticated by ourselves
Mycoplasma contamination	the cells lines tested negative for mycoplasma
Commonly misidentified lines (See ICLAC register)	*Name any commonly misidentified cell lines used in the study and provide a rationale for their use.*

Human research participants

Policy information about studies involving human research participants

Population characteristics	Sampling and analyses were stratified by sex, 10-year age groups (0-9 years to ≥80 years) and region of France.
Recruitment	Participants specimens were collected for routine clinical purposes without potential self-selection.
Ethics oversight	The Ethics Committee (Comité de Protection des Personnes Ile-de-France VI, CHU Pitié-Salpétrière Hospital, Paris, France) waived the need for ethical approval for the collection, analysis and publication of the retrospectively obtained and anonymized specimens and data for this study.

Note that full information on the approval of the study protocol must also be provided in the manuscript.
